# Duplicated network meta-analysis in advanced prostate cancer: a case study and recommendations for change

**DOI:** 10.1186/s13643-022-02137-6

**Published:** 2022-12-16

**Authors:** David J. Fisher, Sarah Burdett, Claire Vale, Ian R. White, Jayne F. Tierney

**Affiliations:** MRC Clinical Trials Unit at UCL, Institute of Clinical Trials and Methodology, Unversity College London, 90 High Holborn, London, WC1V 6LJ UK

**Keywords:** Case study, Scoping review, Network meta-analysis, Research duplication, Published data, Prostate cancer

## Abstract

**Background:**

Research overlap and duplication is a recognised problem in the context of both pairwise and network systematic reviews and meta-analyses. As a case study, we carried out a scoping review to identify and examine duplicated network meta-analyses (NMAs) in a specific disease setting where several novel therapies have recently emerged: hormone-sensitive metastatic prostate cancer (mHSPC).

**Methods:**

MEDLINE and EMBASE were systematically searched, in January 2020, for indirect or mixed treatment comparisons or network meta-analyses of the systemic treatments docetaxel and abiraterone acetate in the mHSPC setting, with a time-to-event outcome reported on the hazard-ratio scale. Eligibility decisions were made, and data extraction performed, by two independent reviewers.

**Results:**

A total of 13 eligible reviews were identified, analysing between 3 and 8 randomised comparisons, and comprising between 1773 and 7844 individual patients. Although the included trials and treatments showed a high degree of overlap, we observed considerable variation between identified reviews in terms of review aims, eligibility criteria and included data, statistical methodology, reporting and inference. Furthermore, crucial methodological details and specific source data were often unclear.

**Conclusions and recommendations:**

Variation across duplicated NMAs, together with reporting inadequacies, may compromise identification of best-performing treatments. Particularly in fast-moving fields, review authors should be aware of all relevant studies, and of other reviews with potential for overlap or duplication. We recommend that review protocols be published in advance, with greater clarity regarding the specific aims or scope of the project, and that reports include information on how the work builds upon existing knowledge. Source data and results should be clearly and completely presented to allow unbiased interpretation.

**Supplementary Information:**

The online version contains supplementary material available at 10.1186/s13643-022-02137-6.

## Background

Research overlap and duplication is a recognised problem in the context of both pairwise and network systematic reviews. It has been estimated that two-thirds of pairwise meta-analyses [1] and over three-quarters of published network meta-analyses (NMAs) have overlap with at least one other, often to a high degree [2]. Commentators have noted that whilst some duplication is justifiable in terms of independent replication, larger-scale duplication brings a risk of confusion and wasted effort [1], which may be heightened in the context of rapidly evolving fields such as COVID-19 [3].

Network meta-analysis is an increasingly influential tool for evidence synthesis, with particular worth in situations where multiple treatments are to be ranked with respect to a common standard-of-care, or where no head-to-head comparison data exists. However, methods for NMA are numerous, and continue to evolve. Hence, research duplication may partly be explained by an ongoing lack of consensus regarding their conduct, particularly choices as to which interventions, trials and data items should be included and compared [2]. This situation persists despite efforts such as the network extension to the PRISMA statement [4] and the emergence of Living Systematic Reviews [5, 6].

For decades, androgen deprivation therapy (ADT) had been the established standard-of-care for hormone-sensitive metastatic prostate cancer (mHSPC). However, recent trials and pairwise meta-analyses [7, 8] have demonstrated improved survival from adding docetaxel or abiraterone acetate to ADT, sparking debate regarding their relative merits [9–11]. Furthermore, there has been a suggestion that response to these treatments may be influenced by a patient subgroup defined as “high-volume” or “high-risk” metastatic disease (HVD [12] or HRD [13]). As a result of our own research in this area, we became aware of multiple NMAs with similar scope but apparently heterogeneous methods and conclusions. Hence, we carried out a scoping review to identify and evaluate research duplication in this setting, and to summarise variations in results and conclusions between reviews. In doing so, we aim to highlight the important issues and make recommendations for future practice.

## Methods

### Literature review

To identify a representative cohort of reviews, we searched systematically (see Additional file 1) for indirect treatment comparisons (ITC), mixed (or multiple) treatment comparisons (MTC) and network meta-analyses (NMA) of systemic treatments in the mHSPC setting. Eligible reviews did not need to be systematic, but must have presented at least one evidence-based inference on an indirect treatment comparison with a time-to-event outcome reported on the hazard-ratio scale. To avoid confusion with more recent therapeutic developments [14, 15], we specifically targeted meta-analyses referencing both “docetaxel” and “abiraterone”, but excluded analyses of “enzalutamide” or “apalutamide”. Searching was performed originally in May 2019, updated in January 2020, within the MEDLINE and EMBASE databases (via the OVID interface) with no restrictions on year of publication or language. Review abstracts were initially screened for study design and disease setting, followed by full-text screening to confirm eligibility. Abstracts from the proceedings of American Society of Clinical Oncology (ASCO) and European Society of Medical Oncology (ESMO) were potentially eligible. If a report was accepted as a conference abstract but subsequently published as a peer-reviewed article, we included both, but extracted data from the article.

### Data extraction

Two independent reviewers (DF and SB) extracted data concerning the timing of completion of the review, estimated by the date submitted for peer review or to conference committee; and of the results entering the public domain, estimated by the date of publication of a peer-reviewed article or conference abstract book. We also extracted data on funding sources, inclusion and exclusion criteria for included trials and patients, definitions of endpoints and of important patient subgroups, and the network HRs themselves together with details of statistical methodology and software used to obtain them. Specifically, we recorded whether common-effect or random-effects modelling was used for the primary analysis, whether under a Bayesian or frequentist statistical framework, details of testing for network inconsistency and heterogeneity, and whether any trial-level factors were adjusted for. Furthermore, we obtained the original source publications for all trials included in eligible reviews, and extracted the reported HRs for relevant endpoints (see Additional files 4 and 5), together with details of statistical methodology used to obtain them. Finally, we assessed each review against the PRISMA-NMA checklist ([4]; see Additional file 6).

### Data analysis

Our primary synthesis was a narrative comparison, across reviews, of aims, scope, methodology, reporting and interpretation, to form an exemplar of the potential extent of NMA duplication. In particular, we aimed to highlight aspects of particular consequence for review quality or interpretation. We also extracted reported hazard ratios for the effect of abiraterone acetate versus docetaxel on time-to-event endpoints, and attempted to recreate the results of each NMA from reported trial results, using Stata v15.1 (StataCorp LP, College Station, TX) and the user-written “network” package [16]. We documented variations in estimated effect size and precision between reviews, and made narrative suggestions for how such variation might be explained by differences in observed review characteristics.

## Results

We identified 19 eligible articles, published between August 2017 and December 2019, describing thirteen individual reviews. Ten reviews [17–26] were reported within peer-reviewed journals, of which five had previously also appeared in the form of one or more conference abstracts [27–32]. Three further reviews [33–35] were described in conference proceedings only. A flow diagram is shown in Additional file 2, and eligible reviews are summarised in Additional file 3.

### Description of relevant reviews

**Fig. 1:**
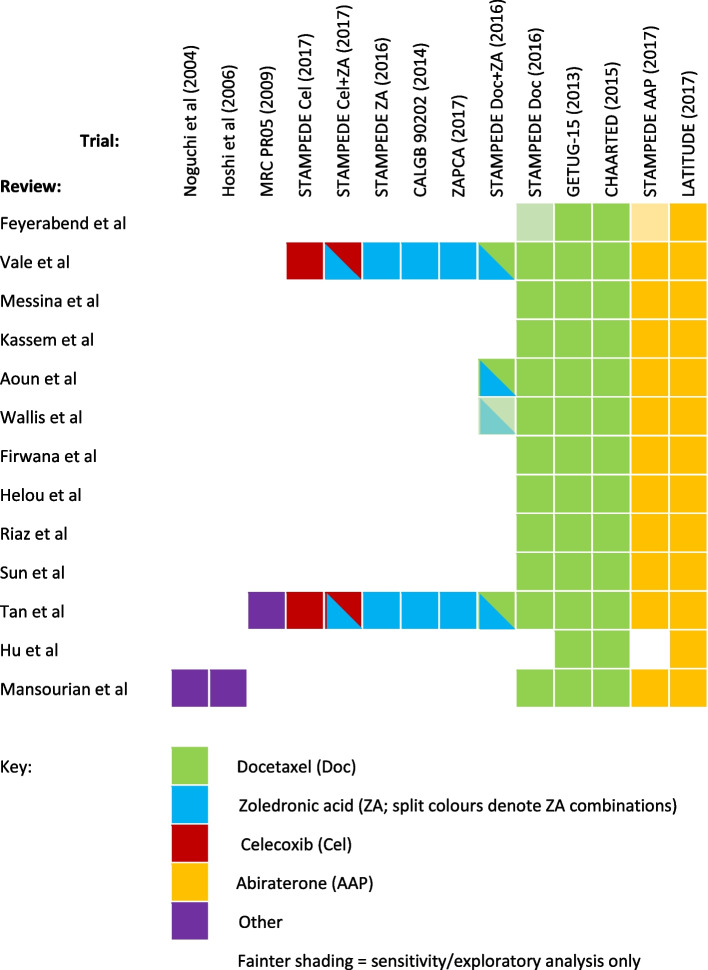
Trials and treatments included in the primary analysis from which an indirect comparison of AAP+ADT vs Doc+ADT may be obtained. Note: Reviews are ordered by earliest known date of submission, acceptance or publication (online or print). Conference abstracts were assumed to be accepted as of the publicised submission deadline. For visual clarity, trials are clustered by included treatments rather than placed in strict order of publication. See Additional files 3, 4 and 5 for details and references for the reviews and trials. Green = docetaxel (Doc); blue = zoledronic acid (ZA); maroon = celecoxib (Cel); amber = abiraterone (AAP); purple = other. Split colours = treatment combinations, as above. Light shading = included in sensitivity/exploratory analysis only

**Fig. 2:**
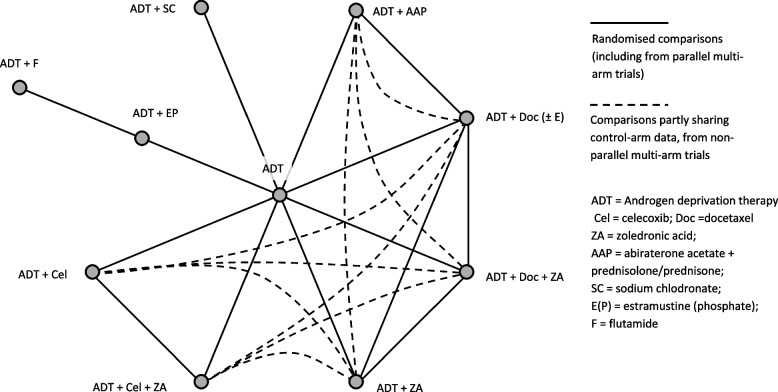
Theoretical full extent of the network

All trials included in eligible reviews investigated the addition of one or more treatments, such as abiraterone, celecoxib, docetaxel, and zoledronic acid, to the standard-of-care of androgen deprivation therapy (ADT) compared to ADT alone, or a combination of these treatments [36, 37]. One large adaptive trial [38] compared multiple research treatments under the same protocol, such that data from 14 randomised comparisons were represented across the reviews from within nine trial protocols. Each review used data from between three and twelve randomised comparisons (Fig. 1), comprising between 1773 and 7844 patients. A matrix of the trials and treatment comparisons from each review is shown in Fig. 1, and the theoretical network resulting from analysis of all such data simultaneously is shown in Fig. 2. The relevant source data from each of the relevant trials is given in Additional files 4 and 5.

### Sources of variation

We observed considerable variation between the included reviews in terms of review aims, eligibility criteria and included data, statistical methodology, reporting and inference.

#### Review aims and funding sources

All 13 eligible reviews either stated or implied an aim to identify optimal treatments for hormone-sensitive prostate cancer. Two reviews stated additional specific aims of including updated results [22] and/or improved methodology [21, 22]. Four aimed to evaluate treatment efficacy within pre-defined patient subgroups [20, 23–25], and four stated the aim of incorporating health economic considerations [25] or adverse effects [18, 23, 35].

Eight of the 13 reviews did not report funding sources or claimed no conflicts of interest, with a further three declaring links to industry but without a direct conflict of interest with any included trials [25, 31, 33, 34]. Of the remaining two reviews, one [24, 27] was directly sponsored by the funders of an included trial [13], with the stated aim of placing that trial in context of a specific patient subgroup. The other [22, 28] shared an academic institution with an included trial [36, 37, 39], although there were no common funding sources external to the institution. Multiple trial investigators were named as co-authors to this review due to the collaborative nature of the project.

#### Included trials

Seven of the 13 reviews described themselves as “systematic” in their title or abstract [20, 22–24, 33–35], and a further 4 [18, 21, 25, 26] described themselves as such at least once elsewhere in their reports. All but one [17] reported that a formal search strategy had been used, although only five [18, 21, 23, 24, 26] referenced the PRISMA guidelines [4] or presented a review flowchart. All reviews specified a disease setting of hormone-sensitive prostate cancer (HSPC), and only included randomised controlled trials (RCTs). Nine reviews [17, 18, 20–23, 25, 33, 34] specified that trials must include a control arm of ADT alone. Of the remainder, only one review [24] included the direct comparison of abiraterone vs docetaxel from the STAMPEDE platform trial, first available as a conference abstract in September 2017 [40] and therefore potentially also eligible for other reviews (see Additional file 3). Eight reviews [17, 19, 21, 22, 26, 33–35] aimed to include trials in metastatic disease (M1). Two further reviews [24, 25] narrowed their target to metastatic high-volume disease (M1 HVD), of which one [24] additionally restricted to *newly diagnosed* (that is, untreated) M1 HVD but presented sensitivity analyses including data from other clinically-relevant trials with broader inclusion criteria. By contrast, three other reviews explicitly broadened their criteria to include trials in the high-risk [20] or locally advanced [18, 23] *non-metastatic* setting, although one [18] ultimately limited their analysis to M1 trials due to lack of data.

#### Included treatments

The set of included treatments varied depending upon the aims of the review. Eight reviews [17, 18, 23–25, 33–35] only included data comparing docetaxel or abiraterone plus ADT to ADT alone, reflecting the focus of clinical interest. Four others included at least one additional treatment combination from the STAMPEDE platform trial [36, 37]. Two such reviews [19, 20] included the zoledronic acid plus docetaxel combination, treating this simply as additional docetaxel data. The remaining two [21, 22] included *all* published results from STAMPEDE where a treatment combination was compared to ADT alone, plus other trials with data on similar comparisons (Fig. 1); and performed network analysis (see “Statistical methods” section, below). One such review [22] gave an explicit justification for the exclusion of one particular treatment (sodium chlodronate), referring to earlier work [7] where the treatment was considered separately due to “differences in mechanisms of action” and because it “is not commonly used in practice”. By contrast, two other treatments rarely used in recent times (estramustin phosphate and flutamide [41, 42]) *were* included, without explicit justification, in a different review [26].

#### Included participants

Patient inclusions were necessarily governed by the reported data from eligible trials. The vast majority of included trials (see Additional files 4 and 5) conformed to the intention-to-treat principle [43]; the exceptions being two small, older trials [26, 41, 42] where small numbers of patients were not analysed due to protocol deviation or non-eligibility.

Some reviews applied additional inclusion criteria within the HSPC setting, most commonly to metastatic disease (M1; see “Included trials” section above). One of the largest relevant trials (STAMPEDE [36, 37, 39]) randomised men both with M1 and high-risk non-metastatic (M0) disease; but many results were reported within patient subgroups such that M1 men could be included within M1-only reviews. However, it was not always clear that review authors extracted or analysed these data consistently. For example, one review [17] specified that only M1 men were eligible, but the reported data suggested that the STAMPEDE result for *all* randomised patients (that is, M0 and M1 combined) had been extracted.

Only two reviews [20, 23] investigated patient subgroups other than M0/M1 or HVD: looking at age, performance status, Gleason score and presence of visceral metastases. Neither used the recommended “deft” approach to testing for subgroup interactions in the meta-analytic context as recommended by Fisher et al. [44].

#### Included outcomes

All reviews focussed on time-to-event outcomes reported on the relative (hazard ratio) scale. Eleven of the 13 reviews reported overall survival (OS) results (Additional file 3), generally thought to be the most clinically relevant outcome in this setting [45] and for which there was a consistent definition across trials and meta-analyses. Ten reviews reported results on intermediate (secondary) outcomes based around the time to disease progression (Additional file 3), but there were notable differences between reviews in how such data were handled. Precise outcome definitions varied between trials, and some trials reported effect sizes for multiple intermediate outcomes. Because of this, one review [20] considered that such data were “not reported consistently enough between trials to allow for pooling”. Three reviews [21, 24, 26] imposed a specific definition of the intermediate outcome, with the aim of maximising consistency but at the risk of trial exclusions and loss of information. By contrast, two reviews [22, 23] argued that intermediate outcome definitions were sufficiently similar as to allow clinical interpretation of the pooled result, selecting the most prominent estimate from trials where more than one definition was used. One such review [22] explicitly reported their observations regarding heterogeneity of definitions, and included a discussion of the potential impact on review conclusions (see “Comparison of primary results and of reviewers’ interpretations” section). The remaining reviews did not provide sufficient information to determine how intermediate outcome data were handled. Additional outcomes were considered by some reviews in accordance with specific review aims (see “Review Aims and Funding Sources” section), but are not within the scope of this case study.

#### Included results

Two of the included trials (see "Included trials" section) reported “long-term” results subsequent to their primary analysis reports, to allow secondary outcomes sufficient time to mature [12, 46–48]. Particularly in a time-to-event context, updated results can increase power and precision by capturing additional events [49]. Although three reviews explicitly stated that data from the most recent available trial report would be used [18, 20, 22], many others were inconsistent or unclear. For example, one review [19] referenced updated results for an included trial [47] but appeared to use an older set of results [46] in their analysis. Updated OS results from another trial were reported in a conference abstract [48], with intermediate outcome results presented at the conference itself. However, only a single review [22] incorporated these results in place of older published results for that trial [12].

#### Statistical methods

A wide range of statistical methods were used. Three reviews [17, 33, 34] simply carried out pairwise meta-analyses of included treatments versus standard-of-care, with inference for indirect comparisons based upon a test of subgroup difference [50]. A more common approach, used in five reviews [18–20, 23, 25], was the “Bucher method” [51], applicable to three-treatment triangular networks but which has been criticised for estimating a separate heterogeneity variance for each comparison [50]. Two reviews [19, 20] accommodated the “docetaxel plus zoledronic acid” comparison from STAMPEDE within such a framework by treating it as an additional docetaxel comparison, reflecting a similar approach sometimes used in pairwise meta-analysis [52]. Four others analysed networks of four or more treatments using mixed treatment comparison (MTC) methods, either using frequentist multivariate analysis [22] or a Bayesian framework [21, 24, 26]. Such methods allow indirect evidence to contribute to effect estimation, which can increase precision [53]. Overall, of the nine frequentist reviews, six used random-effects modelling; one [18] used common-effect modelling; one [19] used a hybrid method (see Additional file 3); and one [25] was unclear. Only one review [22] reported network inconsistency or heterogeneity statistics. No reviews adjusted for any trial-level factors.

Due to its adaptive multi-arm design [38], multiple treatment comparisons from the STAMPEDE trial may be correlated. If a review includes such comparisons as though they were independent trials, double-counting of control arm observations may lead to inflated variances. However, only three reviews [21, 22, 24] explicitly discussed this issue, despite it being highlighted in the PRISMA-NMA statement [4]. One such review [21] stated that “treatment comparisons … from the same study were modelled … with a [Bayesian] correlation prior distributed uniformly on 0–0.95”. Another [22] sought to estimate the correlations themselves using event counts by treatment arm. Both also included zoledronic acid combination arms separately from docetaxel and celecoxib alone, which added strength to the docetaxel network comparison. The remaining review [24] was unique in including direct comparison data from STAMPEDE of abiraterone vs docetaxel [40, 54]. Despite correctly noting “differences in the period of enrolment” between the direct comparison and the original comparisons against ADT, and “uncertainty in the extent of overlap of populations for each of the comparisons” [24], they did not attempt to formally account for this, choosing instead to perform sensitivity analyses.

#### Reporting

Three reviews were reported in conference proceedings only [33–35], and a further two [17, 26] took the form of “letters to the editor” rather than full research articles; understandably, these all conformed poorly to PRISMA-NMA guidelines [4]. Although the eight fully peer-reviewed articles generally conformed better (see Additional file 6), risk-of-bias assessments and handling of multi-arm trials were common omissions, and only two reviews [22, 23] published their protocol in advance. There was also some evidence of outcome reporting bias, for example one review [26] presented an indirect estimate for the intermediate outcome but not for overall survival, despite evidence that both outcomes were analysed. Reporting of source data and description of statistical methodology was often poor, making it difficult to recreate the reported indirect treatment comparisons. Inconsistencies in use of source data, and minor reporting errors such as inconsistent patient or event counts, further hindered attempts to make reasonable judgments as to how such analyses might be recreated.

### Comparison of primary results and of reviewers’ interpretations

**Fig. 3:**
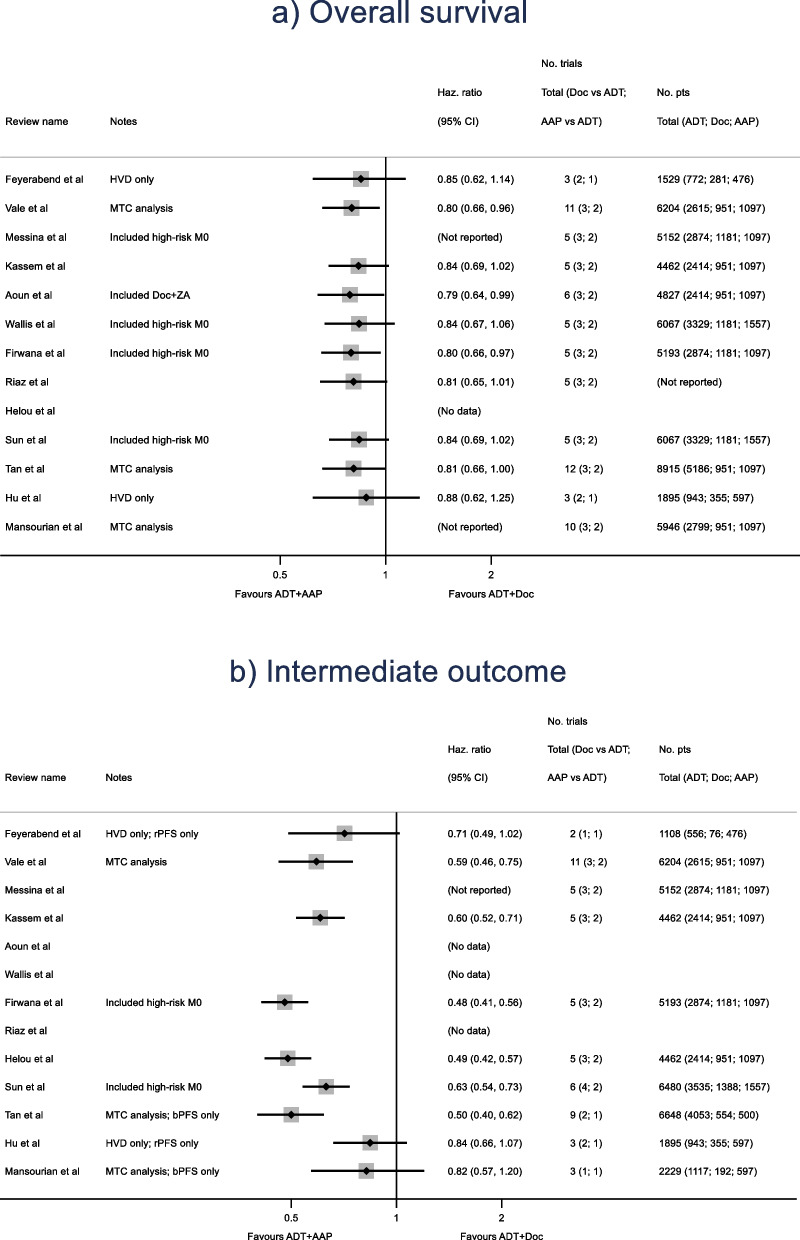
Forest plot of reported indirect comparisons of abiraterone acetate plus standard of care (ADT + AAP) vs docetaxel plus standard of care (ADT + Doc) for (**a**) the overall survival outcome and (**b**) Intermediate outcome. (Not reported) = analysis was performed, but relevant data could not be extracted from the report; (No data) = analysis was not performed; HVD = high volume disease; MTC = multiple treatment comparison; M0 = non-metastatic disease; ZA = zoledronic acid; rPFS = time to disease progression as assessed via radiography, or to death; bPFS = time to disease progression as assessed via PSA measurement, or to death. See Additional file 3 for references for the reviews

Twelve of the 13 reviews analysed overall survival (OS), of which 9 explicitly reported an indirect estimate of abiraterone versus docetaxel. Despite the differences described above, results were fairly similar, with HRs of around 0.80 (range 0.79 to 0.88) and of borderline significance at the 5% level (Fig. 3a). The most obvious discriminating feature was a wider confidence interval from two reviews [24, 25] which reported specifically on the high-volume disease (HVD) sub-population. Overall, eight reviews [17–19, 23, 33, 34] (including three MTC-based reviews [21, 22, 24] and one of the HVD-only reviews [24]) drew tentative conclusions regarding an OS advantage for abiraterone over docetaxel. By contrast, three reviews [20, 25, 35] stated categorically that there was *no* difference in OS; the conclusions for the final review [26] were unclear. Notably, conclusions differed among three reviews including an identical set of trials: two [18, 20] stated explicitly that their analysis did not demonstrate statistical significance, whilst the third [19] stated that “despite several limitations stemming from the paucity of comparative evidence, our results favour [abiraterone] over [docetaxel]”. This would appear to be due to a notable difference in effect size between two reports of the same trial [46, 47] (see “Sources of variation” section), with one review [19] extracting from the earlier report.

Of 10 reviews which analysed an intermediate outcome, seven reported indirect estimates [18, 21–26] and a further two, both conference abstracts [33, 34], reported sufficient details of methods and associated results for the relevant estimates to be accurately recreated. Due to the variations in intermediate outcome definition (see “Sources of variation” section), we took the results most prominently presented or described in each review (see Additional file 3). The estimates here were more varied, with HRs ranging from 0.48 to 0.84 (Fig. 3b). Much of this heterogeneity may be explained by the two HVD-only reviews [24, 25] which reported noticeably smaller effect estimates. Of these, one [24] concluded that a “positive trend” was seen both in overall survival and in the intermediate outcome, whilst the other [25] stated that “no statistically significant difference” was seen. The third non-significant result in Fig. 3b is taken from a review [26] for which descriptions of methodology and source data were particularly limited, and we were unable to recreate their analysis.

In the remaining six reviews [18, 21–23, 33, 34], the intermediate outcome results were all strongly significant at conventional levels, and this was reflected in the reviewers’ conclusions. However, effect size heterogeneity is still apparent in Fig. 3b, with HRs of around 0.60 reported by three reviews [18, 22, 23] and of around 0.50 by three others [21, 33, 34]. One review [22] carried out sensitivity analyses of the choice of intermediate outcome effect from specific trials, and saw results consistent with both effect sizes. Another [21] imposed restrictions on intermediate outcome definitions to improve consistency, excluding two trial results [12, 13] included elsewhere. This *a priori* decision was justified by the review authors, and its potential limitations acknowledged. The remaining observed review-level heterogeneity would appear to be due to one trial [13] reporting two intermediate outcome effect size estimates that differed noticeably from each other (see Additional file 5).

## Discussion

Our scoping review and case study of NMAs analysing treatments for metastatic hormone-sensitive metastatic prostate cancer identified thirteen eligible reviews, demonstrating considerable variation in the aims, data included, statistical and other methodology, reporting and inference.

Overall, most of the eligible reviews had broadly similar objectives, but did not necessarily explain clearly why their approach offered a beneficial or differing perspective. Only two [22, 23] registered protocols in advance with the PROSPERO international prospective register (CRD42017071811 and CRD42017071268, available from https://www.crd.york.ac.uk/prospero). All reviews but one [26] reported within a year of the publication of two major trials showing improved survival with abiraterone [13, 39]. Within the first 6 months alone, four articles were submitted for peer review [17–20] and six conference abstracts [27–29, 33–35] were accepted. Among the former, methodology was relatively simple and, while it may be surmised that speed of dissemination was at least a partial motivation, such reviews may cause confusion if later more in-depth work suggests a differing interpretation. Notably, of nine peer-reviewed articles identified in total, only three made reference to any other reviews in the same field. One [22] did so to highlight methodological advantages, and the other two [23, 24] to demonstrate that they built on previous work in order to clarify specific aspects. It is notable that, despite the observed variation between reviews, the estimated effects of abiraterone versus docetaxel on the definitive OS outcome displayed far less heterogeneity between reviews (Fig. 3a) than did those on intermediate outcomes (Fig. 3b). This suggests that treatment effect heterogeneity in this case study is explained primarily by inconsistencies in included data.

Although duplication and overlap in meta-analysis and NMA have been discussed previously [1, 2], this is to our knowledge the first detailed case study in the NMA setting, answering a previous call for further exploration [2]. Our literature review was comprehensive, and we are not aware of any substantial relevant unpublished data. The most inclusive reviews [21, 22] included over 75% of relevant studies, or arguably 100% if older treatments are disregarded (Fig. 1). This compares to a maximum of just 55% from a previous study of much larger networks of biologics for rheumatoid arthritis [2]. Superficially, the network (Fig. 2) is fairly small and simple, with only one multi-arm trial and no indirect treatment loops. This allowed us to make cleaner and more granular comparisons between NMAs, although we were unable to fully examine issues such as network geometry and “lumping” or “splitting” of nodes [2]. Furthermore, we have been able to highlight the very specific complexities introduced by the inclusion of an adaptive multi-arm trial [38]. Since utilisation of such novel trial designs are on the increase across health care areas [55], there is a corresponding need to identify gaps in review methodology which may risk biased or inefficient results. To broaden understanding of the various issues, we encourage researchers in other clinical settings to undertake similar case studies of duplicated NMAs.

In this case study, duplication resulted primarily from the situation of multiple new mHSPC treatments emerging within a relatively short period of time, raising various unanswered questions. An obvious limitation of all included reviews is their use of aggregate data. An individual participant data NMA is currently being conducted within the STOPCAP M1 programme [56, 57], and should allow many of the issues discussed here to be resolved. As the majority of data were from large-scale randomised controlled trials, we did not attempt to draw any conclusions about the possible effects of trial-level bias on review results. We note also that a project protocol was not published a priori, partly due to the rapid conceptualisation of this project following concerns regarding duplication within an active research field. However, since to our knowledge no similar scoping work exists in this area, we consider the likely impact of this omission to be negligible.

Trials of new life-prolonging therapies continue to produce results in this setting, and a similar situation of duplicated NMAs may already be arising [58–60]. Likewise, respected commentators have noted the risks of duplication in the context of COVID-19 [61], where for example the antiviral drug remdesivir has recently been the focus of multiple reviews [62–64]. Ongoing rapid research into prognosis and treatment of COVID-19 will likely continue to raise similar issues. Living cumulative NMAs [6], an extension to the general concept of living systematic reviews [5], have been proposed as a solution to issues both of research wastage and of network fragmentation (lack of overlap). However, dissemination of such reviews still requires pragmatic decision-making, for example regarding scope or inclusions, with consequent risk of some degree of duplication.

Increased availability of published results, such as via open-access policies and online supplementary data, has many advantages in terms of reducing risk of bias and decentralisation of research efforts from the most developed parts of the world. However, duplication of effort has clear cost and resourcing implications. It has been recommended that existing reviews be identified “as a compulsory first step” [65] in the review process. This advice could be broadened to also encompass potentially eligible *trials*, whether completed or ongoing. These should be identified early, and review authors should thereafter keep track of their progress, particularly in fast-moving fields. Communication with trial investigators is a sometimes-overlooked resource which may help identify additional data and to achieve consensus on ongoing and future research.

## Conclusion

The effectiveness of network and indirect treatment comparison meta-analysis as a tool for identifying best-performing treatments may be compromised by overlap and duplication. Included data, methodology, reporting and interpretation may vary across reviews with similar scope, risking confusion. To mitigate this, we recommend that detailed review protocols be published in advance, following the PRISMA-NMA statement [4]. Review authors should be aware of relevant trials and NMAs at all stages of development, and should provide information on how their work builds upon existing knowledge. Source data and results should be clearly and completely presented to allow unbiased interpretation. In turn, review users may need to be aware of the existence of duplication, and to exercise judgment when utilising review conclusions.

## Supplementary Information

**Additional file 1.** Search Strategy for literature review.

**Additional file 2.** PRISMA Flow diagram showing results of systematic literature search.

**Additional file 3.** Summary of characteristics of included reviews.

**Additional file 4.** Published trial data used in reviews of overall survival.

**Additional file 5.** Published trial data used in reviews of intermediate composite outcomes.

**Additional file 6.** PRISMA NMA assessments for each included review.

## Data Availability

All data generated or analysed during this study are included in this published article and its supplementary information files.

## Abbreviations

NMA: Network meta-analysis
mHSPC: Metastatic hormone-sensitive prostate cancer
ADT: Androgen deprivation therapy
HVD: High-volume disease
HRD: High-risk disease
ITC: Indirect treatment comparison
MTC: Mixed treatment comparison
M1: Metastatic disease
M0: Non-metastatic disease
OS: Overall survival

## References

[1] Siontis KC, Hernandez-Boussard T, Ioannidis JP (2013). Overlapping meta-analyses on the same topic: survey of published studies. BMJ.

[2] Naudet F, Schuit E, Ioannidis JPA (2017). Overlapping network meta-analyses on the same topic: survey of published studies. Int J Epidemiol.

[3] Clarke M (2020). BMJ Opinion.

[4] Hutton B, Salanti G, Caldwell DM, Chaimani A, Schmid CH, Cameron C (2015). The PRISMA extension statement for reporting of systematic reviews incorporating network meta-analyses of health care interventions: checklist and explanations. Ann Intern Med.

[5] Elliott JH, Turner T, Clavisi O, Thomas J, Higgins JP, Mavergames C (2014). Living systematic reviews: an emerging opportunity to narrow the evidence-practice gap. PLoS Med.

[6] Vandvik PO, Brignardello-Petersen R, Guyatt GH (2016). Living cumulative network meta-analysis to reduce waste in research: a paradigmatic shift for systematic reviews. BMC Med.

[7] Vale CL, Burdett S, Rydzewska LH, Albiges L, Clarke NW, Fisher D (2016). Addition of docetaxel or bisphosphonates to standard of care in men with localised or metastatic, hormone-sensitive prostate cancer: a systematic review and meta-analyses of aggregate data. Lancet Oncol.

[8] Rydzewska LHM, Burdett S, Vale CL, Clarke NW, Fizazi K, Kheoh T (2017). Adding abiraterone to androgen deprivation therapy in men with metastatic hormone-sensitive prostate cancer: a systematic review and meta-analysis. Eur J Cancer.

[9] Bilgin B, Sendur MA, Hizal M, Akinci MB, Sener Dede D, Yalcin B (2017). Docetaxel or abiraterone in addition to androgen deprivation therapy in metastatic castration-sensitive prostate cancer. Future Oncol.

[10] McNamara M, Sweeney C, Antonarakis ES, Armstrong AJ (2018). The evolving landscape of metastatic hormone-sensitive prostate cancer: a critical review of the evidence for adding docetaxel or abiraterone to androgen deprivation. Prostate Cancer Prostatic Dis.

[11] Sharma AP, Mavuduru RS, Bora GS, Devana SK, Singh SK, Mandal AK (2018). STAMPEDEing metastatic prostate cancer: CHAARTing the LATITUDEs. Indian J Urol.

[12] Sweeney CJ, Chen YH, Carducci M, Liu G, Jarrard DF, Eisenberger M (2015). Chemohormonal therapy in metastatic hormone-sensitive prostate cancer. N Engl J Med.

[13] Fizazi K, Tran N, Fein L, Matsubara N, Rodriquez-Antolin A, Alekseev BY (2017). Abiraterone plus prednisone in metastatic castration-sensitive prostate cancer. N Engl J Med.

[14] Chi KN, Agarwal N, Bjartell A, Chung BH, de Santana P, Gomes AJ, Given R (2019). Apalutamide for metastatic, castration-sensitive prostate cancer. N Engl J Med.

[15] Davis ID, Martin AJ, Stockler MR, Begbie S, Chi KN, Chowdhury S (2019). Enzalutamide with standard first-line therapy in metastatic prostate cancer. N Engl J Med.

[16] White IR (2015). Network meta-analysis. Stata J.

[17] Messina C, Messina M, Boccardo F (2018). Abiraterone or docetaxel for castration-sensitive metastatic prostate cancer? That is the question!. Eur Urol.

[18] Kassem L, Shohdy KS, Abdel-Rahman O (2018). Abiraterone acetate/androgen deprivation therapy combination versus docetaxel/androgen deprivation therapy combination in advanced hormone-sensitive prostate cancer: a network meta-analysis on safety and efficacy. Curr Med Res Opin.

[19] Aoun F, El Rassy E, Sleilaty G, Assi T, Bakouny Z, Kattan J (2017). The optimal treatment of metastatic hormone-naive prostate cancer: abiraterone acetate or docetaxel?. Future Oncol.

[20] Wallis CJD, Klaassen Z, Bhindi B, Goldberg H, Chandrasekar T, Farrell AM (2017). Comparison of abiraterone acetate and aocetaxel with androgen deprivation therapy in high-risk and metastatic hormone-naive prostate cancer: a systematic review and network meta-analysis. Eur Urol.

[21] Tan PS, Aguiar P, Haaland B, Lopes G (2018). Addition of abiraterone, docetaxel, bisphosphonate, celecoxib or combinations to androgen-deprivation therapy (ADT) for metastatic hormone-sensitive prostate cancer (mHSPC): a network meta-analysis. Prostate Cancer Prostatic Dis.

[22] Vale CL, Fisher DJ, White IR, Carpenter J, Burdett S, Clarke NW (2018). What is the optimal systemic treatment for men with metastatic, hormone- naive prostate cancer? A STOPCAP systematic review and network meta-analysis. Ann Oncol.

[23] Sun G, Zhang X, Chen J, Liao B, Liu Z, Zhao J (2018). What kind of patients with castration-naive prostate cancer can benefit from upfront docetaxel and abiraterone: A systematic review and a network meta-analysis. Urol Oncol.

[24] Feyerabend S, Saad F, Li T, Ito T, Diels J, Van Sanden S (2018). Survival benefit, disease progression and quality-of-life outcomes of abiraterone acetate plus prednisone versus docetaxel in metastatic hormone-sensitive prostate cancer: a network meta-analysis. Eur J Cancer.

[25] Hu X, Qu S, Yao X, Li C, Liu Y, Wang J (2019). Abiraterone acetate and docetaxel with androgen deprivation therapy in high-volume metastatic hormone-sensitive prostate cancer in China: An indirect treatment comparison and cost analysis. Cost Eff Resour Alloc.

[26] Mansourian M, Ghasemi K, Khorsandi D, Vaseghi G. Comparative effectiveness of all available treatments for metastatic hormone-sensitive prostate cancer: a network meta-analysis. Am J Ther. 2020;27(5):e541–3. 10.1097/MJT.0000000000001008 31107252

[27] Feyerabend S, Saad F, Li T, Ito J, Diels J, van Sanden P (2017). Indirect comparison of abiraterone acetate and docetaxel for treatment of metastatic “hormone-sensitive” prostate cancer. Ann Oncol.

[28] Vale CL, Fisher DJ, Carpenter J, White IR, Burdett S, Clarke NW (2017). What are the optimal systemic treatments for men with metastatic, hormone-sensitive prostate cancer? A STOPCaP systematic review and network meta-analysis. Ann Oncol.

[29] Sun G, Zhang X, Chen J, Liao B, Zhao J, Shen P (2018). Is it the time to shift paradigms in castration naive prostate cancer (CNPC): a metaanalysis of upfront docetaxel and abiraterone in men with CNPC. J Clin Oncol.

[30] Aguiar P, Tan P, Simko S, Barreto C, Aguiar B, Del Giglio A (2018). Network metanalysis and cost-effectiveness of abiraterone, docetaxel or placebo plus androgen deprivation therapy (ADT) for hormone-sensitive advanced prostate cancer. J Clin Oncol.

[31] Hu X, Yao X, Li C, Liu Y, Xiong T, Qu S (2018). Comparison of abiraterone acetate and docetaxel with androgen deprivation therapy in high-volume metastatic hormone-sensitive prostate cancer (mHSPC): an updated network meta-analysis and cost minimization analysis in China. J Clin Oncol.

[32] Feyerabend S, Saad F, Ito T, Diels J, Van Sanden S, de Porre P (2019). Overall survival with abiraterone acetate plus prednisone vs. docetaxel for the treatment of metastatic hormone-sensitive prostate cancer: an updated network meta-analysis. Eur Urol.

[33] Firwana B, Sonbol M, Mahmoud F, Arnaoutakis K (2018). Treatments for metastatic hormone-sensitive prostate cancer: a systematic review. J Clin Oncol.

[34] Helou J, Catton C, Bauman G, Fazelzad R, Raphael J (2018). Abiraterone or docetaxel in men with metastatic castration-sensitive prostate cancer: a pooled analysis of castration resistance-free survival and toxicity. J Clin Oncol.

[35] Riaz I, Almutairi A, Ali Z, Alhifany A, Bhattacharjee S, Abraham I (2018). Abiraterone acetate (AA) or docetaxel (D) in metastatic castration-sensitive prostate cancer (mCSPC): A systematic review and network meta-analysis of randomized clinical trials (RCTs). J Clin Oncol.

[36] James ND, Sydes MR, Clarke NW, Mason MD, Dearnaley DP, Spears MR (2016). Addition of docetaxel, zoledronic acid, or both to first-line long-term hormone therapy in prostate cancer (STAMPEDE): survival results from an adaptive, multiarm, multistage, platform randomised controlled trial. Lancet.

[37] Mason MD, Clarke NW, James ND, Dearnaley DP, Spears MR, Ritchie AW (2017). Adding celecoxib with or without zoledronic acid for hormone-naïve prostate cancer: long-term survival results from an adaptive, multiarm, multistage, platform, randomized controlled trial. J Clin Oncol.

[38] Sydes MR, Parmar MK, Mason MD, Clarke NW, Amos C, Anderson J (2012). Flexible trial design in practice - stopping arms for lack-of-benefit and adding research arms mid-trial in STAMPEDE: a multi-arm multi-stage randomized controlled trial. Trials.

[39] James ND, de Bono JS, Spears MR, Clarke NW, Mason MD, Dearnaley DP (2017). Abiraterone for prostate cancer not previously treated with hormone therapy. N Engl J Med.

[40] Sydes MR, Mason MD, Spears MR, Clarke NW, Dearnaley D, Ritchie AWS (2017). Adding abiraterone acetate plus prednisolone (AAP) or docetaxel for patients (pts) with high-risk prostate cancer (PCa) starting long-term androgen deprivation therapy (ADT): Directly randomised data from STAMPEDE (NCT00268476). Ann Oncol.

[41] Noguchi M, Noda S, Yoshida M, Ueda S, Shiraishi T, Itoh K (2004). Chemohormonal therapy as primary treatment for metastatic prostate cancer: a randomized study of estramustine phosphate plus luteinizing hormone-releasing hormone agonist versus flutamide plus luteininzing hormone-releasing hormone agonist. Int J Urol.

[42] Hoshi S, Yamaguchi O, Fujioka T, Arai Y, Tomita Y, Habuchi T (2006). A randomized comparative study of endocrine monotherapy and a combination of estramustine phosphate with the endocrine therapy in patients with untreated stage D prostate cancer. Int J Clin Oncol.

[43] Hollis S, Campbell F (1999). What is meant by intention-to-treat analysis? Survey of published randomised controlled trials. BMJ.

[44] Fisher DJ, Carpenter JR, Morris TP, Freeman SC, Tierney JF (2017). Meta-analytical methods to identify who benefits most from treatments: daft, deluded, or deft approach?. BMJ.

[45] ICECaP working Group (2015). The development of Intermediate Clinical Endpoints in Cancer of the Prostate (ICECaP). J Natl Cancer Inst.

[46] Gravis G, Fizazi K, Joly F, Oudard S, Priou F, Esterni B (2013). Androgen-deprivation therapy alone or with docetaxel in non-castrate metastatic prostate cancer (GETUG-AFU 15): a randomised, open-label, phase 3 trial. Lancet Oncol.

[47] Gravis G, Boher JM, Joly F, Soulie M, Albiges L, Priou F (2016). Androgen Deprivation Therapy (ADT) Plus Docetaxel Versus ADT Alone in Metastatic Non castrate Prostate Cancer: Impact of Metastatic Burden and Long-term Survival Analysis of the Randomized Phase 3 GETUG-AFU15 Trial. Eur Urol.

[48] Sweeney C, Chen Y, Liu G, Carducci M, Jarrard DF, Eisenberger M (2016). Long term efficacy and QOL data of chemohormonal therapy (C-HT) in low and high volume hormone naïve metastatic prostate cancer (PrCa): E3805 CHAARTED trial. Ann Oncol.

[49] Tierney JF, Stewart LA, Ghersi D, Burdett S, Sydes MR (2007). Practical methods for incorporating summary time-to-event data into meta-analysis. Trials.

[50] Salanti G (2012). Indirect and mixed-treatment comparison, network, or multiple-treatments meta-analysis: many names, many benefits, many concerns for the next generation evidence synthesis tool. Res Synthesis Methods.

[51] Bucher HC, Guyatt GH, Griffith LE, Walter SD (1997). The results of direct and indirect treatment comparisons in meta-analysis of randomized controlled trials. J Clin Epidemiol.

[52] Chemoradiotherapy for Cervical Cancer Meta-analysis Collaboration (2008). Reducing uncertainties about the effects of chemoradiotherapy for cervical cancer: a systematic review and meta-analysis of individual patient data from 18 randomized trials. J Clin Oncol.

[53] Cooper NJ, Peters J, Lai MC, Juni P, Wandel S, Palmer S (2011). How valuable are multiple treatment comparison methods in evidence-based health-care evaluation?. Value Health.

[54] Sydes MR, Spears MR, Mason MD, Clarke NW, Dearnaley DP, de Bono JS (2018). Adding abiraterone or docetaxel to long-term hormone therapy for prostate cancer: directly randomised data from the STAMPEDE multi-arm, multi-stage platform protocol. Ann Oncol.

[55] Blagden SP, Billingham L, Brown LC, Buckland SW, Cooper AM, Ellis S (2020). Effective delivery of Complex Innovative Design (CID) cancer trials-A consensus statement. Br J Cancer.

[56] Tierney JF, Vale CL, Parelukar WR, Rydzewska L, Halabi S (2019). Evidence synthesis to accelerate and improve the evaluation of therapies for metastatic hormone-sensitive prostate cancer. Eur Urol Focus.

[57] Rogozińska E, Vale C, Fisher D, Rydzewska L, Burdett S, White I (2019). Systematic review and individual participant data meta-analyses of systemic treatments for hormone-sensitive metastatic prostate cancer.

[58] Di Nunno V, Santoni M, Mollica V, Conti A, Montironi R, Battelli N (2020). Systemic treatment for metastatic hormone sensitive prostate cancer: a comprehensive meta-analysis evaluating efficacy and safety in specific sub-groups of patients. Clin Drug Investig.

[59] Marchioni M, Di Nicola M, Primiceri G, Novara G, Castellan P, Paul A (2019). New anti-androgen compounds compared to docetaxel in metastatic hormone sensitive prostate cancer: results from a network meta-analysis. J Urol.

[60] Sathianathen N, Koschel S, Thangasamy I, Teh J, Alghazo O, Butcher G (2020). Indirect comparisons of efficacy between combination approaches in metastatic hormone-sensitive prostate cancer: a systematic review and network meta-analysis. Eur Urol.

[61] Clarke M. How can we avoid research waste during the covid-19 pandemic and plan for the future? In: BMJ Opinion; 2020 [cited 2020 Nov 06]. Available from: https://blogs.bmj.com/bmj/2020/04/21/mike-clarke-avoid-research-waste-covid-19-pandemic-planfuture/.

[62] Nasir M, Talha KA, Islam T, Saha SK, Selina F, Parveen RA (2020). Use of remdesivir in the management of COVID-19: a systematic review on current evidences. Mymensingh Med J.

[63] Musa A, Pendi K, Hashemi A, Warbasse E, Kouyoumjian S, Yousif J (2020). Remdesivir for the treatment of COVID-19: a systematic review of the literature. Western J Emerg Med.

[64] Frediansyah A, Nainu F, Dhama K, Mudatsir M, Harapan H (2021). Remdesivir and its antiviral activity against COVID-19: a systematic review. Clin Epidemiol Glob Health.

[65] Moher D. The problem of duplicate systematic reviews. BMJ. 2013;347:f5040. 10.1136/bmj.f5040 23945367

