# Supplementary material for: Duplicated network meta-analysis in advanced prostate cancer: a case study and recommendations for change
Source: Syst Rev. 2022 Dec 16;11:274. doi: 10.1186/s13643-022-02137-6 (PMC9755764; doi:10.1186/s13643-022-02137-6)
Supplement: Supplementary file 1 — Additional file 1. Search Strategy for literature review. [file 13643_2022_2137_MOESM1_ESM.docx]

# Additional file 1: Search Strategy for literature review

Database: Embase Classic+Embase <1947 to 2020 January 31>, Ovid MEDLINE(R) and Epub Ahead of Print, In-Process & Other Non-Indexed Citations, Daily and Versions(R) <1946 to January 31, 2020>

1 (prostate adj5 cancer).mp. [mp=ti, ab, hw, tn, ot, dm, mf, dv, kw, fx, dq, nm, kf, ox, px, rx, ui, sy]

2 ((hormone or castration) adj5 (naive or sensitive)).mp. [mp=ti, ab, hw, tn, ot, dm, mf, dv, kw, fx, dq, nm, kf, ox, px, rx, ui, sy]

3 (metastatic or advanced or "high risk").mp. [mp=ti, ab, hw, tn, ot, dm, mf, dv, kw, fx, dq, nm, kf, ox, px, rx, ui, sy]

4 (indirect* adj5 compar*).mp. [mp=ti, ab, hw, tn, ot, dm, mf, dv, kw, fx, dq, nm, kf, ox, px, rx, ui, sy]

5 (multiple adj5 comparison).mp. [mp=ti, ab, hw, tn, ot, dm, mf, dv, kw, fx, dq, nm, kf, ox, px, rx, ui, sy]

6 (network adj5 analys*).mp. [mp=ti, ab, hw, tn, ot, dm, mf, dv, kw, fx, dq, nm, kf, ox, px, rx, ui, sy]

7 (docetaxel and abiraterone).mp. [mp=ti, ab, hw, tn, ot, dm, mf, dv, kw, fx, dq, nm, kf, ox, px, rx, ui, sy]

8 meta-analys*.mp. [mp=ti, ab, hw, tn, ot, dm, mf, dv, kw, fx, dq, nm, kf, ox, px, rx, ui, sy]

9 meta-regress*.mp. [mp=ti, ab, hw, tn, ot, dm, mf, dv, kw, fx, dq, nm, kf, ox, px, rx, ui, sy]

10 (pool* adj5 analys*).mp. [mp=ti, ab, hw, tn, ot, dm, mf, dv, kw, fx, dq, nm, kf, ox, px, rx, ui, sy]

11 1 and 2 and 3

12 4 or 5 or 6

13 8 or 9 or 10

14 7 and 13

15 12 or 14

16 11 and 15
