# Supplementary material for: Duplicated network meta-analysis in advanced prostate cancer: a case study and recommendations for change
Source: Syst Rev. 2022 Dec 16;11:274. doi: 10.1186/s13643-022-02137-6 (PMC9755764; doi:10.1186/s13643-022-02137-6)
Supplement: Supplementary file 2 — Additional file 2. PRISMA Flow diagram showing results of systematic literature search. [file 13643_2022_2137_MOESM2_ESM.docx]

# Additional file 2: PRISMA Flow diagram showing results of systematic literature search

Records identified from search

(n=82)

Abstracts screened

after removing duplicates

(n=57)

Irrelevant records excluded

(n=12)

Details:

Castrate-resistant disease setting (n=5)

Non-statistical review article (n=4)

Randomised trial (n=1)

Methodological study (n=1)

Biomarker analysis (n=1)

Articles assessed for eligibility

(n=45)

Articles excluded (n=26)

Details:

Abiraterone or docetaxel not included (n=12)

Enzalutamide or apalutamide included (n=5)

Letter; no original data (n=4)

Unable to obtain article; abstract suggests

no original work eligible for review (n=3)

No survival outcomes (n=1)

No indirect comparison (n=1)

Articles included in review

(n=19; describing 13 unique reviews)

Peer-reviewed articles

(n=10)

Conference abstracts

(n=9)
