# Supplementary material for: Duplicated network meta-analysis in advanced prostate cancer: a case study and recommendations for change
Source: Syst Rev. 2022 Dec 16;11:274. doi: 10.1186/s13643-022-02137-6 (PMC9755764; doi:10.1186/s13643-022-02137-6)
Supplement: Supplementary file 3 — Additional file 3. Summary of characteristics of included reviews. [file 13643_2022_2137_MOESM3_ESM.docx]

# Additional file 3: Summary of characteristics of included reviews

Note: Ordered by the earliest known date of submission, acceptance or publication (online or print). Conference abstracts were assumed to be accepted as of the publicised submission deadline.

| **Name** | **Earliest known date submitted or accepted, and published** | **Number of treatments**  **(including ADT);**  **eligible* trials;**  **reported* patients** | **Outcomes considered** | **Primary statistical methodology and software** |
| --- | --- | --- | --- | --- |
| Feyerabend et al^24 27^ | Accepted as conference abstract May 2017  Peer-reviewed article available online Sep 2018 | Treatments = 3  Trials = 3 (NDx HVD only)  Patients = 1,773 (NDx HVD only)  Exploratory analysis  including STAMPEDE:  Treatments = 3  Trials = 5  Patients = 3,861 | OS  Radiographic PFS (rPFS)  = time to radiographic progression  or any death | Bayesian Multiple Treatment Comparison;  WinBUGS (Lunn DJ et al. Stat Comput 2000;10:325-37) |
| Vale et al^22 28^ | Accepted as conference abstract May 2017  Peer-reviewed article available online Feb 2018 | Treatments = 7  Trials = 11  Patients = 6,204 | OS  Failure-Free Survival (FFS)  = time to PSA failure, clinical progression or any death | Multiple Treatment Comparison using random-effects (REML) multivariate meta-analysis;  Stata (StataCorp LP, College Station, TX) |
| Messina et al^17^ | Accepted Jul 2017  Available online Aug 2017 | Treatments = 3  Trials = 5  Patients = 5,152 | Overall survival (OS)  Progression-Free Survival  (PFS; definition unclear) | Random-effects pairwise; non-statistical indirect comparison  Cochrane Review Manager (The Nordic Cochrane Centre, Copenhagen) |
| Kassem et al^18^ | Submitted Aug 2017  Available online Feb 2018 | Treatments = 3  Trials = 9 (incl. M0)  Patients = 7,469 (incl. M0)  Primary analysis of M1:  Treatments = 3  Trials = 5  Patients = 4,462 | OS  PFS; definition unclear | Indirect Treament Comparison  using common-effect;  MetaXL (EpiGear, Sunrise Beach, Queensland, Australia) |
| Aoun et al^19^ | Submitted Sep 2017  Available online Nov 2017 | Treatments = 4  Trials = 6  Patients = 4,827 | OS only | Generalised Indirect Treatment Comparison using IVHet (Doi SA et al. *Contemp Clin Trials* 2015; 45: 130-8); MetaXL (ibid) |
| Wallis et al^20^ | Accepted Oct 2017  Available online Oct 2017 | Treatments = 3  Trials = 5  Patients = 6,067  Exploratory analysis  including Doc+ZA:  Treatments = 4  Trials = 6  Patients = 7,844 | OS only | Indirect Treatment Comparison  using random-effects;  MetaXL (ibid) |
| Firwana et al^33^ | Accepted as conference abstract Oct 2017  Conference proceedings available Feb 2018 | Treatments = 3  Trials = 5  Patients = 5,193 | OS  FFS (definition unclear) | Random-effects pairwise; test for subgroup difference |
| Helou et al^34^ | Accepted as conference abstract Oct 2017  Conference proceedings available Feb 2018 | Treatments = 3  Trials = 5  Patients = 4,462 | Castration resistance-free survival  (CFS) only; definition unclear | Random-effects pairwise; test for subgroup difference |
| Riaz et al^35^ | Accepted as conference abstract Oct 2017  Conference proceedings available Feb 2018 | Treatments = 3  Trials = 5  Patients = Not reported | OS only | Bayesian Indirect Treatment Comparison; WinBUGS (ibid) |
| Sun et al^23 29^ | Accepted as conference abstract Feb 2018  Peer-reviewed article available online Oct 2018 | Treatments = 3  Trials = 6  Patients = 6,480 | OS  FFS: time to PSA failure,  clinical progression or any death | Indirect Treatment Comparison using both common- and random-effects; CADTH ITC (Wells GA et al. “Indirect Evidence: Indirect Treatment Comparisons in Meta-Analysis”. Canadian Agency for Drugs and Technologies in Health, Ottawa) |
| Tan et al^21 30^ | Submitted Jan 2018  Available online Jun 2018 | Treatments = 7  Trials = 12  Patients = 8,915 | OS  FFS = time to biochemical progression,  PSA failure, radiographic progression,  clinical progression, or any death | Bayesian Multiple Treatment Comparison; RJags; R (R Foundation for Statistical Computing, Vienna) |
| Hu et al^25 31^ | Accepted as conference abstract Feb 2018  Peer-reviewed article available online Dec 2019 | Treatments = 3  Trials = 3  Patients = 1,895 (HVD only) | OS  PFS (definition unclear, but included results suggest “time to radiographic progression or any death”) | Random-effects pairwise;  Cochrane Review Manager (ibid)  Indirect Treatment Comparison using Microsoft Excel (Microsoft, Richmond, WA) |
| Mansourian et al^26^ | Available online May 2019 (no further info) | Treatments = 7  Trials = 10  Patients = 5,946 | OS  Biochemical PFS (definition unclear) | Bayesian and frequentist Multiple Treatment Comparison; details unclear; software not reported |

(G)ITC = (generalised) indirect treatment comparison^50^; MTC = mixed treatment comparison; MVMA = multivariate meta-analysis

* Some reviews had a broader or more complex scope than mHSPC, and/or included sensitivity or exploratory analyses which narrowed or broadened the primary scope. This table gives the numbers of treatments, trials and patients for the primary scope as given in the text, with additional information where appropriate.
