# Supplementary material for: Duplicated network meta-analysis in advanced prostate cancer: a case study and recommendations for change
Source: Syst Rev. 2022 Dec 16;11:274. doi: 10.1186/s13643-022-02137-6 (PMC9755764; doi:10.1186/s13643-022-02137-6)
Supplement: Supplementary file 4 — Additional file 4. Published trial data used in reviews of overall survival. [file 13643_2022_2137_MOESM4_ESM.docx]

# Additional file 4: Published trial data used in reviews of overall survival

| **Trial name** | **Source reference**  **Identifier (year)** | **Treatment *** | **Treatment**  **n/N** | **Control**  **n/N** | **HR (95% CI)** |
| --- | --- | --- | --- | --- | --- |
| Noguchi et al | PMID: 14706014 (2004) | ADT + EP  vs ADT + F | 14/29 | 11/22 | Log rank p=0.80 |
| Hoshi et al | PMID: 16937304 (2006) | ADT + EP | -/31 | -/26 | Log rank p=0.040 in favour of EP |
| MRC PR05 | PMID: 19674936 (2009) | ADT + SC | 132/140 | 126/138 | 0.77 (0.60, 0.98) |
| GETUG 15 | PMID: 23306100 (2013) | ADT + Doc | 88/192 | 88/193 | 1.01 (0.75, 1.36) |
|  | PMID: 26610858 (1016) | ADT + Doc (LTFU) | 115/192 | 127/193 | 0.88 (0.68, 1.14) |
|  |  | ADT + Doc (LTFU, HVD) | -/92 | -/91 | 0.78 (0.56, 1.09) |
| CALGB 90202 | PMID: 24590644 (2014) | ADT + ZA | 134/323 | 151/322 | 0.88 (0.70, 1.12) |
| CHAARTED | PMID: 26244877 (2015) | ADT + Doc | 101/397 | 136/393 | 0.61 (0.47, 0.80) |
|  | DOI: 10.1093/annonc  /mdw372.04 (2016) | ADT + Doc (LTFU) | 188/397 | 211/393 | 0.72 (0.59, 0.89) |
|  |  | ADT + Doc  (LTFU, HVD) | 137/263 | 162/250 | 0.63 (0.50, 0.79) |
| STAMPEDE M1 | PMID: 26719232 (2016) | ADT + Doc | 144/362 | 350/724 | 0.76 (0.62, 0.92) |
|  |  | ADT+ZA+Doc | 158/365 | 350/724 | 0.79 (0.66, 0.96) |
|  |  | ADT + ZA | 170/366 | 350/724 | 0.93 (0.77, 1.11) |
|  | PMID: 28300506 (2017) | ADT + Cel | 110/188 | 245/377 | 0.94 (0.75, 1.18) |
|  |  | ADT+ZA+Cel | 104/190 | 245/377 | 0.78 (0.62, 0.98) |
|  | PMID: 28578639 (2017) | ADT + AAP | 150/500 | 218/502 | 0.61 (0.49, 0.75) |
|  | PMID: 29529169 (2018) | ADT + AAP  vs ADT + Doc | 89/227 | 38/115 | 1.13 (0.77, 1.66) |
| ZAPCA | PMID: 27614621 (2017) | ADT + ZA | -/109 | -/110 | 0.78 (0.49, 1.23) |
| LATITUDE | PMID: 28578607 (2017) | ADT + AAP | 169/597 | 237/602 | 0.62 (0.51, 0.76) |
|  | PMID: 30218976 (2018) | ADT + AAP (HVD) | -/476 | -/479 | 0.57 (0.46, 0.71) |

* versus ADT alone except for Noguchi et al (PMID: 14706014) and the STAMPEDE M1 direct comparison of ADT+AAP vs ADT+Doc (PMID: 29529169).

ADT = androgen deprivation therapy; Doc = docetaxel; ZA = zoledronic acid; AAP = abiraterone acetate; Cel = celecoxib; SC = sodium clodronate; EP = estramustine phosphate; F = flutamide; LTFU = long-term follow-up data; HVD = restricted to “high volume of disease” patient subgroup
