# Supplementary material for: Duplicated network meta-analysis in advanced prostate cancer: a case study and recommendations for change
Source: Syst Rev. 2022 Dec 16;11:274. doi: 10.1186/s13643-022-02137-6 (PMC9755764; doi:10.1186/s13643-022-02137-6)
Supplement: Supplementary file 5 — Additional file 5. Published trial data used in reviews of intermediate composite outcomes. [file 13643_2022_2137_MOESM5_ESM.docx]

# Additional file 5: Published trial data used in reviews of intermediate composite outcomes

| **Trial name** | **Source reference identifier (year)** | **Treatment *** | **Outcome name:**  **components** | **Treatment**  **n/N** | **Control**  **n/N** | **HR (95% CI)** |
| --- | --- | --- | --- | --- | --- | --- |
| GETUG 15 | PMID: 23306100 (2013) | ADT + Doc | bPFS: PSA, clinP, boneP, D | -/192 | -/193 | 0.72 (0.57, 0.91) |
|  | PMID: 26610858 (2016) | ADT + Doc (LTFU) | bPFS: PSA, radP, D | -/192 | -/193 | 0.67 (0.54, 0.84) |
|  |  | ADT + Doc (LTFU, HVD) | rPFS: radP, D | -/92 | -/91 | 0.61 (0.44, 0.83) |
| CALGB 90202 | PMID: 24590644 (2014) | ADT + ZA | PFS: PSA, boneP, D | 211/323 | 230/322 | 0.89 (0.74, 1.07) |
| GETUG 12 | PMID: 26028518 (2015) | ADT + Doc + E | RFS: PSA, clinP, radP, D | 88/207 | 111/206 | 0.71 (0.54, 0.94) |
| CHAARTED | PMID: 26244877 (2015) | ADT + Doc | CRPC: PSA, clinP, radP | -/397 | -/393 | 0.61 (0.51, 0.72) |
|  | DOI: 10.1093/annonc  /mdw372.04 * | ADT + Doc (LTFU) | CRPC: PSA, clinP, radP | 257/397 | 303/393 | 0.61 (0.52, 0.73) |
|  |  | ADT + Doc (LTFU, HVD) | cPFS: boneP, clinP | 142/263 | 176/250 | 0.53 (0.42, 0.67) |
| STAMPEDE M1 | PMID: 26719232 (2016) | ADT + Doc | FFS: PSA, clinP, DPc | 252/362 | 585/724 | 0.61 (0.53, 0.71) |
|  |  | ADT + ZA + Doc | FFS: PSA, clinP, DPc | 254/365 | 585/724 | 0.60 (0.52, 0.69) |
|  |  | ADT + ZA | FFS: PSA, clinP, DPc | 286/366 | 585/724 | 0.89 (0.78, 1.03) |
|  | PMID: 28300506 (2017) | ADT + Cel | FFS: PSA, clinP, DPc | -/188 | -/377 | 0.86 (0.71, 1.04) |
|  |  | ADT + ZA + Cel | FFS: PSA, clinP, DPc | -/190 | -/377 | 0.77 (0.63, 0.93) |
|  | PMID: 28578639 (2017) | ADT + AAP | FFS: PSA, clinP, DPc | 210/500 | 393/502 | 0.31 (0.26, 0.37) |
|  | PMID: 29529169 (2018) | ADT+AAP vs  ADT+Doc | FFS: PSA, clinP, DPc | 109/227 | 79/115 | 0.56 (0.42, 0.75) |
| ZAPCA | PMID: 27614621 (2017) | ADT + ZA | TTF: PSA, clinP, boneS, D | 94/109 | 98/110 | 0.75 (0.57, 1.00) |
| LATITUDE | PMID: 28578607 (2017) | ADT + AAP | rPFS: radP, D | 239/597 | 354/602 | 0.47 (0.39, 0.55) |
|  |  |  | PSA | -/597 | -/602 | 0.30 (0.26, 0.35) |
|  | PMID: 30218976 (2018) | ADT + AAP (HVD) | rPFS: radP, D | -/476 | -/479 | 0.43 (0.36, 0.52) |

* versus ADT alone except for the STAMPEDE M1 direct comparison of ADT+AAP vs ADT+Doc (PMID: 29529169).

ADT = androgen deprivation therapy; Doc = docetaxel; ZA = zoledronic acid; AAP = abiraterone acetate; Cel = celcoxib; SC = sodium clodronate; E = estramustine; LTFU = long-term follow-up data; HVD = restricted to “high volume of disease” patient subgroup

FFS = failure-free survival; PFS = progression-free survival; RFS = relapse-free survival; TTF = time to treatment failure

bPFS = biological PFS; rPFS = radiographic PFS; CRPC = castrate-resistatant prostate cancer

PSA = progression on basis of PSA count; clinP = clinical progression; radP = radiographic progression; boneP = progression of bone disease; boneS = worsening of bone-related symptoms; D = death from any cause; DPC = death from prostate cancer

* ESMO 2016 abstract; data from poster presentation obtained by request from trial investigators
